# Heavy-metal detectors based on modified ferrite nanoparticles

**DOI:** 10.3762/bjnano.9.69

**Published:** 2018-02-28

**Authors:** Urszula Klekotka, Ewelina Wińska, Elżbieta Zambrzycka-Szelewa, Dariusz Satuła, Beata Kalska-Szostko

**Affiliations:** 1 Institute of Chemistry, University of Białystok, Ciołkowskiego 1K, 15-245, Białystok Poland; 2 Faculty of Physics, University of Białystok, Ciołkowskiego 1L, 15-245, Białystok, Poland

**Keywords:** ferrite nanoparticles, heavy metal detection, materials characterization, water purification

## Abstract

In this work, we analyze artificial heavy-metal solutions with ferrite nanoparticles. Measurements of adsorption effectiveness of different kinds of particles, pure magnetite or magnetite doped with calcium, cobalt, manganese, or nickel ions, were carried out. A dependence of the adsorption efficiency on the composition of the inorganic core has been observed. Ferrites surfaces were modified by phthalic anhydride (PA), succinic anhydride (SA), acetic anhydride (AA), 3-phosphonopropionic acid (3-PPA), or 16-phosphohexadecanoic acid (16-PHDA) to compare the adsorption capability of the heavy metals Cd, Cu and Pb. The obtained nanoparticles were structurally characterized by transmission electron microscopy (TEM), X-ray diffraction (XRD), Fourier transform infrared spectroscopy (FTIR) and Mössbauer spectroscopy. The amounts of Cd, Cu and Pb were measured out by atomic absorption spectroscopy (AAS) and energy dispersive X-ray (EDX) as comparative techniques. The performed study shows that SA linker appears to be the most effective in the adsorption of heavy metals. Moreover, regarding the influence of the composition of the inorganic core on the detection ability, the most effective ferrite Mn_0.5_Fe_2.5_O_4_ was selected for discussion. The highest heavy-metal adsorption capability and universality was observed for SA as a surface modifier.

## Introduction

Many research reports show that magnetic nanoparticles can be widely used in medicine for drug delivery, implants manufacture, as components of contrast agents in magnetic resonance imaging (MRI) as well as active centers in hyperthermia treatment [1]. The use of magnetic nanoparticles in drug delivery allows for a significant reduction of the amount of applied medications [2]. Also, one can imagine sensors based on nanostructures possessing very high sensitivity towards particular species due to the high surface area and specific reactivity [3–4]. However, very often not only surface termination but also core composition is of crucial importance for the required functionality.

In food industry or water purification, nanotechnology has begun to play a non-negligible role. However, to date it is considered to be a relatively new application area. Currently, nanostructures are mostly used to improve the quality of food, prolong its storage life, detect contaminants [5] and receive intelligent packaging [6]. A lot of research is focused on the reduction of the amount of fat in the products through the use of nanotechnological solutions. The idea to use fat in the forms of nanoemulsions, e.g., in cream or mayonnaise, became very popular in a novel production process [7]. Moreover, many studies are carried out on sensors based on nanostructures in the detection and removal of toxins, chemical compounds and other pathogens from food. Some nanosensors are designed to improve food–body assimilation by blocking cholesterol or allergens, which are frequently encountered in groceries [8]. Nanomaterials are also widely tested as constituents in the production of modern functional packaging. Packages enriched by nanostructures are not only stronger but also often become "intelligent" and sometimes they can fix themselves after a slight damage. Changing the package color informs that food is not suitable for consumption because either its expiry date passed or packed edibles started to emit harmful compounds [9].

Recent studies show that nanoparticles can be widely used as heavy-metal detectors. Among others, silver [10–11], gold [12] and also magnetic nanoparticles [13–14] usually doped with other elements (e.g., Ca, Mn) [15–16] have been tested for this purpose. Therefore, detailed studies on adsorption efficiency on doped magnetite nanoparticles are very interesting and innovative in order to understand the importance of core composition and surface modification.

The aim of the study is to examine the efficiency of adsorption of heavy metals in artificial solutions on doped magnetite nanoparticles (Ca, Co, Mn, Ni) surface-modified with PA, SA, AA, 3-PPA or 16-PHDA linkers.

## Experimental

### Reagents and solutions

Chemicals used in this work were of analytical grade and they were used without any purification. FeCl_2_·4H_2_O, FeCl_3_·6H_2_O, NH_3_ (25%), CaCl_2_ (anhydrous), MnCl_2_ (anhydrous), NiCl_2_ (anhydrous), CuSO_4_ (anhydrous), PbCl_2_ (anhydrous), Cd(NO_3_)_2_·4H_2_O, and acetic anhydride (AA) were purchased from Polish Chemical Reagents (POCH). CoCl_2_ (anhydrous), tetrabutylammonium hydroxide (TBAOH) (40% in water), phthalic anhydride (PA), 3-phosphonopropionic acid (3-PPA), 16-phosphonohexadecanoic acid (16-PHDA) and phosphate-buffered saline (PBS) were received from Sigma-Aldrich.

#### Apparatus

The structure and morphology of nanoparticles used in the detection experiments were analyzed by X-ray diffraction (XRD) (Agilent Technologies SuperNova diffractometer with micro-focused Mo Kα_2_ (λ = 0.713067 Å) radiation and transmission electron microscopy (TEM) (FEI Tecnai G2 X-TWIN 200 kV microscope). For TEM imaging the nanoparticles after dissolution in ethanol were drop-cast on a carbon-covered 400 mesh Cu grid. The XRD apparatus requires the placement of a small amount of powder on a nylon loop coated with high-viscosity synthetic oil. Infrared spectra (IR) were collected in a spectral range between 400 and 4000 cm^−1^ and were attained by a Nicolet 6700 spectrometer working in transmission mode as a series of 32 repetitive runs. Here, a small amount of powder was directly placed and squeezed into a diamond window for measurement. Mössbauer spectra (MS) were obtained in constant acceleration mode with a ^57^Co in Cr matrix source. The spectra were calibrated using α-Fe foil at room temperature. MS absorbers were prepared by mixing a proper amount of particles with BN filler to obtain 1 cm^2^ tablets.

The amount of Pb, Cu, and Cd elements in the tested solutions was measured by atomic absorption spectrometry (AAS). Measurements were performed using a high-resolution continuum-source atomic absorption spectrometer ContrAA 700 (Analytik Jena AG, Jena, Germany) equipped with a flame atomizer (burner length: 100 mm). The optical system comprises a continuum light source 300 W high-pressure xenon short-arc lamp XBO 301 (GLE, Berlin, Germany) operating in a ”hot-spot” mode, suitable for all the determination of all elements in the wavelength range from 185 to 900 nm. An air–acetylene flame was used for the determination of Pb, Cd, and Cu under optimized conditions, namely a) Pb: burner height: 7 mm, air–C_2_H_2_ flow rate: 75 L·h^−1^; b) Cu: burner height: 4 mm, air–C_2_H_2_ flow rate: 65 L·h^−1^; c) Cd: burner height: 5 mm, air–C_2_H_2_ flow rate: 55 L·h^−1^. Absorbance signals of all analytes were obtained using three pixels (central pixel ± 1) for each element. All absorbance values are the mean values based on three repetitive measurements. The dynamic background correction technique with reference was used. The quantitative determination of Pb, Cd, and Cu was carried out by the external calibration graph technique.

#### Synthesis of ferrite nanoparticles doped with Ca^2+^, Co^2+^, Mn^2+^, or Ni^2+^

To obtain doped ferrite nanoparticles, a modified co-precipitation synthesis of iron(II) and iron(III) chlorides was used [17–19]. As a reference, pure magnetite nanoparticles were also synthesized by the method described below.

In the first step of the synthesis, into each of two three-necked flasks, 0.5% NH_3_ solution was placed and deoxygenated with argon for 20 min. Then, TBAOH was injected to each flask and one solution was heated and stirred (40 °C, 20 min) while the other one was deoxygenated at room temperature. In the next step, a proper amount of FeCl_3_·6H_2_O was added to the heated up flask, and FeCl_2_·4H_2_O to the other one. After 15 min, FeCl_2,_ CaCl_2_, CoCl_2_, MnCl_2_, or NiCl_2_, were additionally added to the second flask in respective amounts. The resultant solution was finally poured into the flask with FeCl_3_·6H_2_O, and the whole mixture was kept at 80 °C for 40 min. Thereafter, the solution was cooled down, removed from the supernatant and washed with deoxygenated acetone. Rinsing was repeated two more times and, finally, the sample was dried by rotary evaporation until a powder was obtained [20–21].

#### Modification of nanoparticles with phthalic anhydride, succinic anhydride and acetic anhydride

The application of Ca ferrite nanoparticles modified with SiO_2_ and phthalic anhydride for the detection of Pb in water and food products was described in the literature [15]. The role of the selected inorganic core, however, was not discussed. The modified nanoparticles used in this study had a selectivity for Pb ions twice of that for other metals. In this paper, we will compare the efficiency of PA with other proposed linkers. For this purpose, every type of prepared ferrite nanoparticles was modified with PA, SA and AA. For the modification solutions of PA, SA or AA in ethanol with a concentration of 100 mM were added to about 8 mg of nanoparticles and stirred for 4 h at room temperature. Then, the functionalized nanoparticles were separated from the solutions using a permanent magnet, washed three times with ethanol and left overnight for drying.

#### Modification of nanoparticles with 3-PPA and 16-PHDA

**Figure 1:**
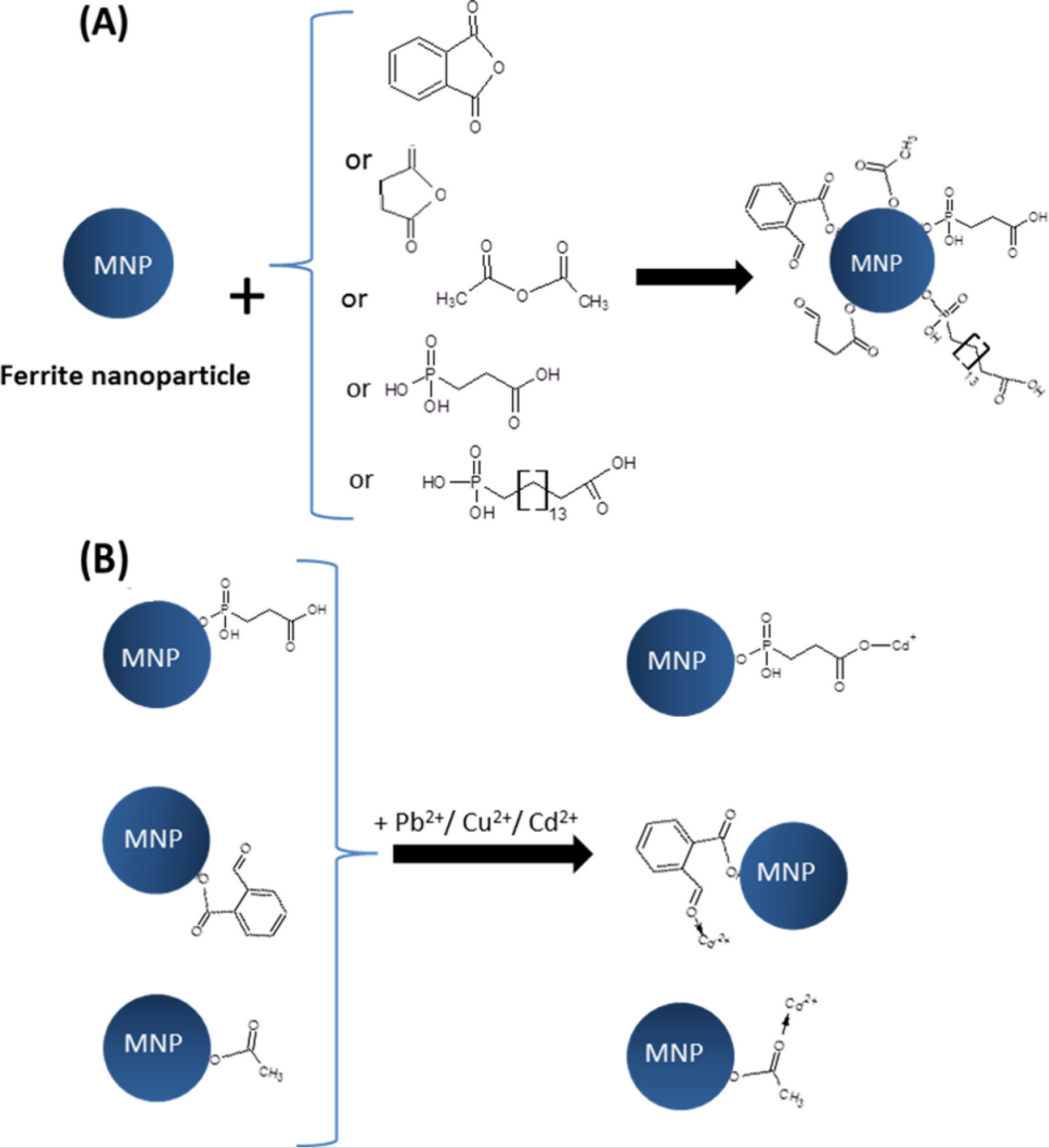
(A) Schematic presentation of the attachment of selected anhydrides and organophosphorus acids to the nanoparticles surface; (B) adsorption of Cu^2+^, Cd^2+^ or Pb^2+^ to the nanoparticles surface via linkers (B).

Another group of compounds tested and selected for the studies as potential heavy-metal detectors are 3-phosphonopropionic acid (3-PPA) and 16-phosphonohexadecanoic (16-PHDA). Before the modification with 3-PPA or 16-PHDA, nanoparticles were washed firstly in acetone and then ethanol, and placed for about 30 s in an ultrasonic bath to ensure proper particles separation. In the next step, 1 mL of 1 mM solution of 3-PPA or 16-PHDA, respectively, were added to the nanoparticles and left at room temperature for 18 h. After this time the mixture was placed in an ultrasonic bath for 1 min, then the supernatant was removed and the nanoparticles were washed three times with PBS and allowed to dry at room temperature [22]. Figure 1A presents the proposed most probable models of linker adsorption. However, the authors do not exclude other configurations.

#### Attachment of Pb^2+^, Cu^2+^ and Cd^2+^ ions

In order to verify if modified nanoparticles are able to detect/select some ions from liquids, aqueous model solutions were prepared. The solutions and particles were analyzed by IR spectroscopy, EDX and AAS measurements after the tests.

For this purpose, approx. 8 mg of surface-modified nanoparticles were added to a 0.1 M aqueous solution of Pb^2+^, Cu^2+^ and Cd^2+^. This mixture was stirred for 10 min by a magnetic stirrer. After this time, the liquid was removed and analyzed by AAS to determine the amount of remaining elements. Nanoparticles were left to dry for 4 h at room temperature. A proposed scheme for the binding of heavy metal ions is presented in Figure 1B.

## Results and Discussion

### Transmission electron microscopy

**Figure 2:**
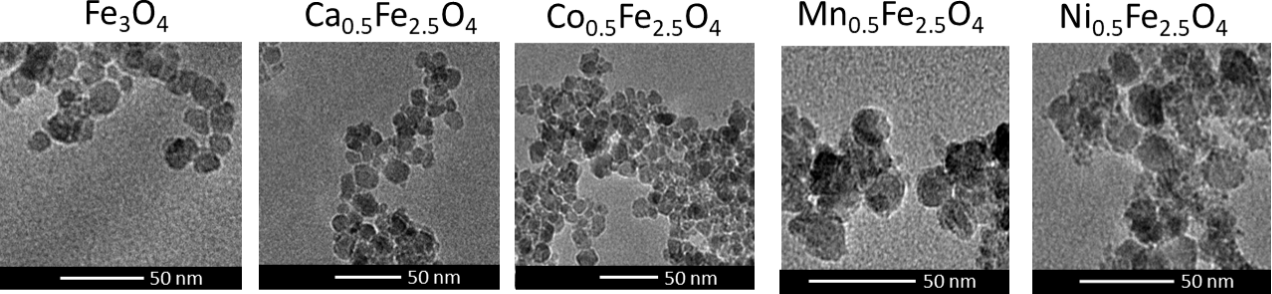
TEM images of prepared ferrite nanoparticles.

Prior to any modification of the nanoparticles, their morphology and structural quality was determined. The most informative method is transmission electron microscopy, by which the morphology of the prepared ferrite nanoparticles was analyzed before surface modification. The obtained images for each kind of fabricated ferrites are presented in Figure 2.

The TEM images show that regardless of doping (Ca^2+^, Co^2+^, Mn^2+^ or Ni^2+^), the studied nanoparticles exhibit a similar round shape and average size. In Table 1, diameters of every type of synthesized nanoparticles estimated from TEM images are collected. The obtained average particle sizes are close to those calculated for pure magnetite nanoparticles, which implies that proposed ions do not disturb the crystallization process and used synthesis constituents fulfill the same growth regimes [23].

**Table 1:** Table with average crystallite sizes of ferrite nanoparticles estimated from TEM images sizes and calculated from the XRD data.

type of nanoparticles	nanoparticle size [nm] (TEM)	average crystallite size [nm] (XRD)

Fe_3_O_4_	13 ± 2	13 ± 2
Ca_0.5_Fe_2.5_O_4_	10 ± 2	13 ± 2
Co_0.5_Fe_2.5_O_4_	13 ± 2	13 ± 2
Mn_0.5_Fe_2.5_O_4_	12 ± 2	10 ± 2
Ni_0.5_Fe_2.5_O_4_	14 ± 2	12 ± 2

### X-ray diffraction

**Figure 3:**
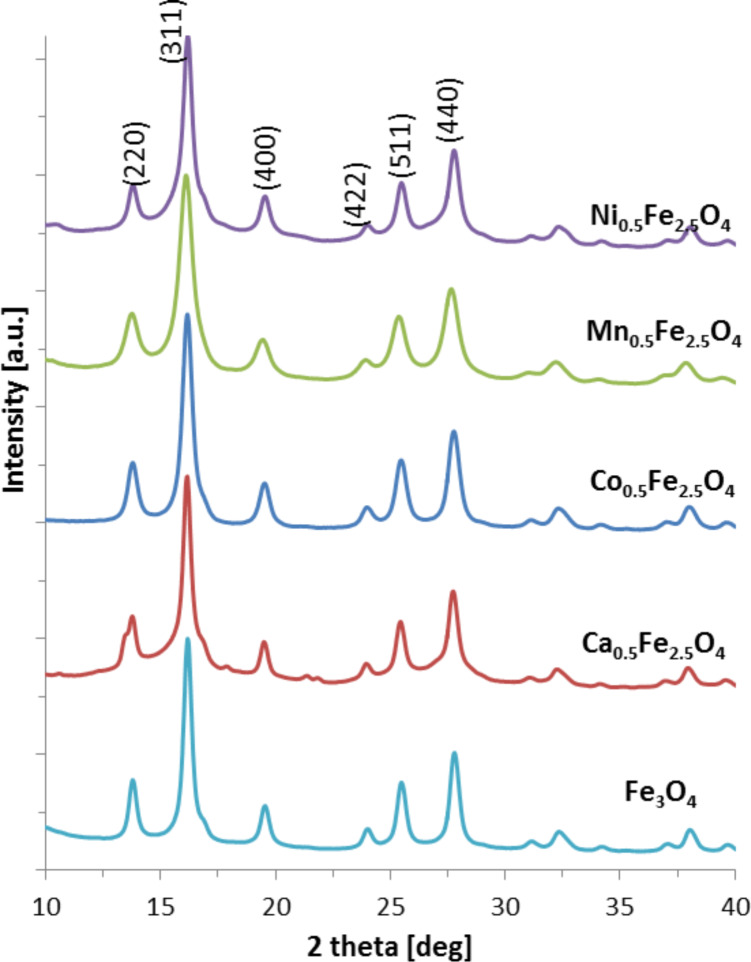
XRD patterns of ferrite nanoparticles.

The fabrication of nonstochiometric compounds affects crystal growth, which is reflected in the crystalization degree. Therefore, examination of the crystallinity of the ferrite nanoparticles was performed by X-ray diffraction. The presence of well-defined sharp patterns proves the incorporation of Ca, Co, Mn and Ni elements into the primary magnetite structure. The obtained diffractograms are depicted in Figure 3.

The XRD patterns show peaks typical for magnetite regardless of the nature of added elements. The 2θ values and the relative intensities unequivocally state the presence of dominate magnetite phase. The most intense reflexes were recognized and indexed to (220), (311), (400), (422), (511), (440), respectively, typical for magnetite [24]. The peak widths, however, are different among the presented samples. This can be caused by chemical modification of the crystallites related to the different ionic radii of the added elements and a resulting slight distortion of the crystal phase. Substitution can take place as a random distribution of particular atoms or as ordered process. Such changes in the structure will cause different modifications of the XRD patterns. The estimation of an average diffraction zone which is related to the crystal size and, therefore, the particle diameter can be calculated by the Scherrer equation (Equation 1). The obtained values are given together with TEM values in Table 1.

[1]
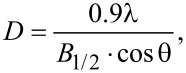


where *D* is the grain size [Å], λ is the wavelength (here 0.7136 Å), *B*_1/2_ is the full width at half maximum intensity of the (311) peak [rad], and Θ is the diffraction angle [rad]. The average crystallite sizes of nanoparticles presented in Table 1 are similar to each other within the error margin. At the same time, the obtained results of the average grain size estimated on the basis of the TEM images agree with the values calculated from XRD. A numerical analysis of XRD patterns also allows for the assessment of crystal cell parameters, which can be found in Table 2. There, literature bulk values are compared with our findings and both are in satisfactory agreement, which also confirms the successful fabrication of requested structures.

**Table 2:** Lattice constants (theoretical and calculated from diffraction patterns) [25–29].

	theoretical [Å]	experimental [Å] ±0.05

γ-Fe_2_O_3_	8.34	8.36
Fe_3_O_4_	8.39	
Ca_0.5_Fe_2.5_O_4_	—	8.37
Co_0.5_Fe_2.5_O_4_	8.38	8.43
Mn_0.5_Fe_2.5_O_4_	8.51	8.40
Ni_0.5_Fe_2.5_O_4_	8.33	8.36

### Infrared spectroscopy

**Figure 4:**
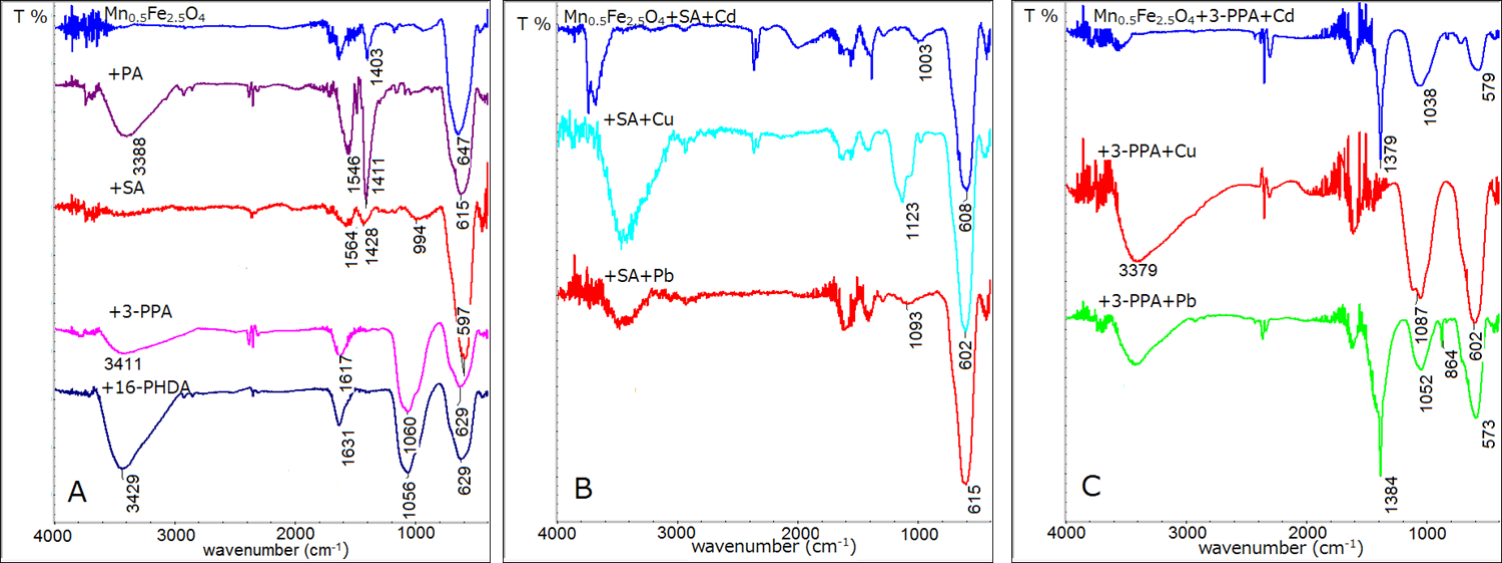
The IR spectra of Mn_0.5_Fe_2.5_O_4_ nanoparticles A) with various linkers used in the experiment, B) with attached SA and Cd, Cu and Pb; and C) with attached 3-PPA and Cd, Cu and Pb.

All samples were measured by IR spectroscopy to see if the surface of nanoparticles changes after core modification and surface functionalization. This method was also expected to serve as a reference for the detection of heavy metals. However, the obtained results show that it is not sufficient in every case. Selected results of modified Mn_0.5_Fe_2.5_O_4_ with different linkers and after the detection of Cd^2+^, Cu^2+^ or Pb^2+^ are presented in Figure 4. This core composition was selected as representative sample for the following tests. Nevertheless, all types of particles were analyzed in the same way.

Figure 4A shows the IR spectra of ferrite nanoparticles before and after linker modification. It can be seen that spectra after modification with PA and SA have increased signals (in comparison to unmodified one) in the range of 1393–1591 cm^−1^, which indicates the presence of C–C bonds in the rings of PA and SA [30]. The spectra after functionalization with 3-PPA or 16-PHDA show the presence of signals around 1060 and 1105 cm^−1^, which proves the presence of P–O and P–O–Fe stretching bonds [31]. Bands at 650–590 cm^−1^ are typical for Fe–O in magnetite/maghemite [32]. This observation confirms a successful modification of the surface of the nanoparticles. The spectra in Figure 4B were collected after the exposition of nanoparticles modified with SA to Cd, Cu and Pb ions. As a result, signals in the range of 1003–1123 cm^−1^ appear in addition to those previously observed. In the last series (Figure 4C) the presence of sharp signals at 1379–1384 cm^−1^ in case of Cd and Pb attachment can be found. In this region modifications in the reference system were also observed after interaction with heavy metal [15]. Therefore, the origin of these signals is most probable due to heavy ion adsorption. However, due to a lack of reference information, it is the only speculation on the origin of appearing new signals.

### Raman spectroscopy

**Figure 5:**
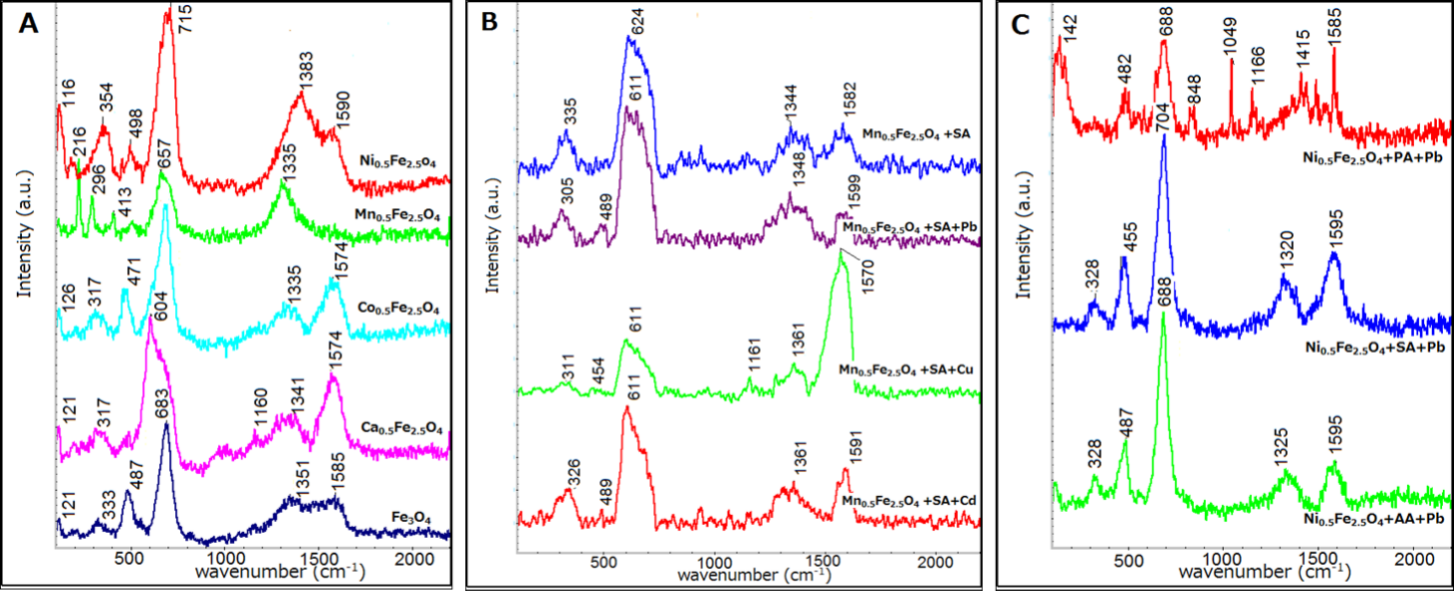
Raman spectra of A) ferrite nanoparticles; B) SA-modified nanoparticles with attached heavy-metal ions; C) various anhydride-modified nanoparticles with attached Pb ions.

After every step (synthesis, surface modification, and heavy metal attachment) powdered samples of nanoparticles were analyzed using Raman spectroscopy giving additional information to IR spectroscopy. In Figure 5 selected spectra are presented.

The Raman spectra show a set of peaks that are typical for inorganic cores (magnetite, maghemite/hematite) with accordingly modified organic shells. In Figure 5A only spectra of unmodified particles are collected. One can see that these spectra are dominated by signals centered around 600–700 cm^−1^. This signal can be attributed both to magnetite and maghemite. The position closer to the middle value of 673–683 cm^−1^ suggests a magnetite-dominated contribution to the signal, while deviation to lower or higher values indicates more maghemite-like contributions [33]. The presence of signals below 670 cm^−1^ implies surface oxidation to maghemite or hematite (for details see Table 3). Signals at a wave number higher than 700 cm^−1^ indicate the presence of an organic shell. Co-doped ferrite nanoparticles have an evidentially dominating peak moved to 600 cm^−1^, which is in good agreement with the doublet observed in Mössbauer spectra, which, in turn, suggests the presence of maghemite. Ni ferrite also shows a similar trend, which is a hint that local Fe surrounding becomes maghemite-like.

**Table 3:** Positions of the most intensive lines in the Raman spectra of tested samples [34].

peak position [cm^−1^]	assignment

524, 673–683	Fe_3_O_4_
322–335, 704–715	γ-Fe_2_O_3_
224, 604	α-Fe_2_O_3_
1335–1383	C–C aliphatic chain/γ-Fe_2_O_3_
1049, 1166, 1415, 1585	C–C, aromatic ring chain vibrations

In Figure 5B, the spectra of SA-modified Mn_0.5_Fe_2.5_O_4_ nanoparticles after the adsorption of different ions are depicted. The comparison of spectra collected in Figure 5B with unmodified particles (Figure 5A) indicates that surface functionalization by SA causes the presence of a band at 1592 cm^−1^, and that the adsorption of heavy-metal ions causes further alternation of the signals in the range of 1200–1700 cm^−1^, which is connected to the interaction of the metal ions with SA and the nanoparticles. A shift of IR signals after heavy-metal adsorption was reported previously [15].

Figure 5C shows the modulation of Raman spectra by different compounds at the surface. A number of additional bands seen in case of PA are due to its specific chemical structure (Table 3). Considering all above-mentioned findings, Raman spectroscopy confirms the surface modification of the nanoparticles and the attachment of heavy-metal ions.

### Mössbauer spectroscopy

**Figure 6:**
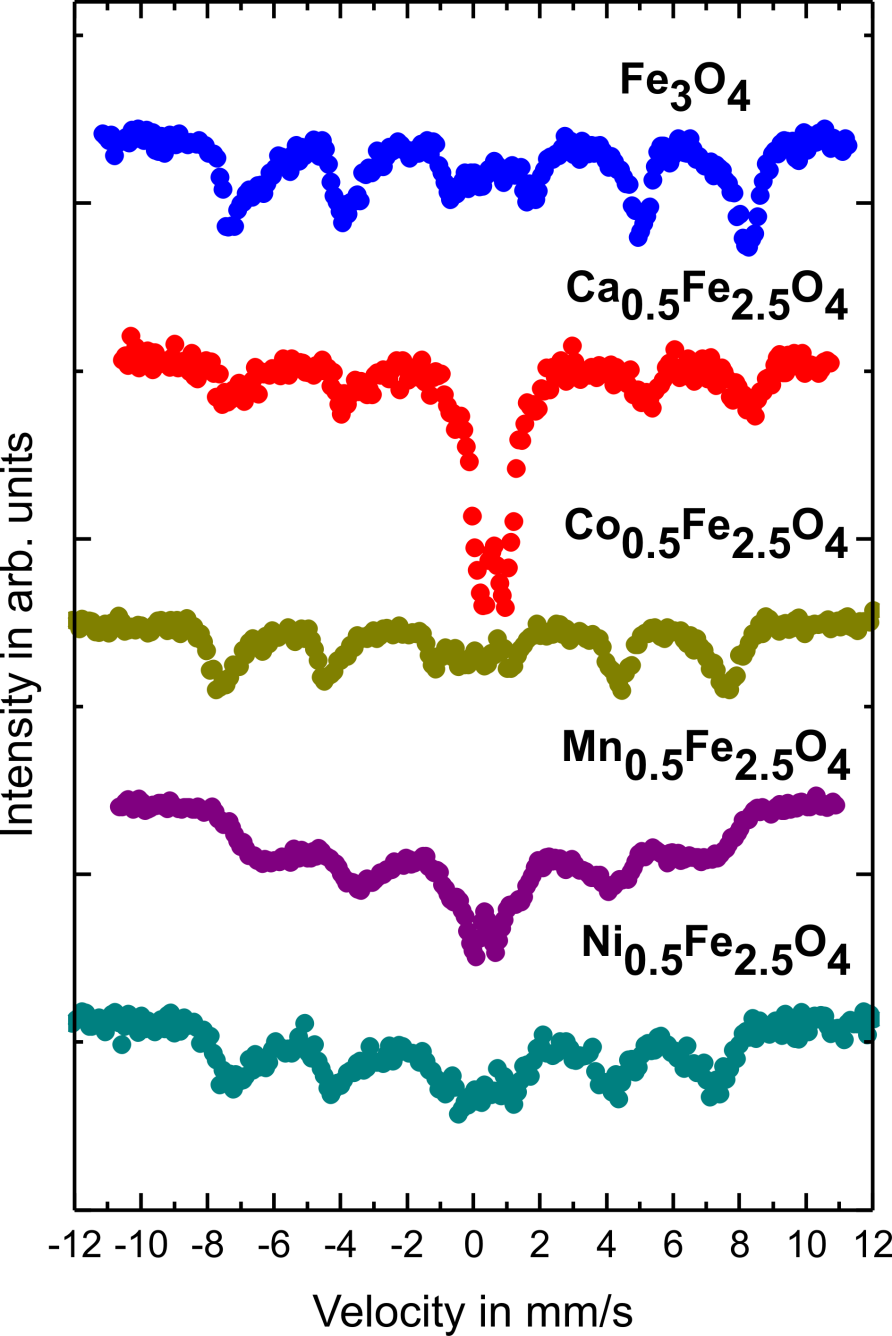
Mössbauer spectra of Fe_3_O_4_, Ca_0.5_Fe_2.5_O_4_, Co_0.5_Fe_2.5_O_4_, Mn_0.5_Fe_2.5_O_4_ and Ni_0.5_Fe_2.5_O_4_ nanoparticles.

The magnetic properties of obtained ferrite nanoparticles were examined by Mössbauer spectroscopy at room temperature. The obtained spectra are presented in Figure 6. All spectra show mostly the same features. The first one is the presence of a sextet with broadened lines, which is actually a superposition of sextets corresponding to Fe atoms at the A and B sites of the magnetite structure [35]. The broadening depends on the type of dopant atoms. In the case of Co ions, the full width at half maximum of the spectral lines is much smaller than in the other cases. An especially wide spectrum is observed in the case of magnetite doped by Mn atoms. The value of the average hyperfine magnetic field on the iron atoms is highest on the sample with Co dopant. The second characteristic feature is the presence of a doublet in the central part of the spectrum, which is connected to the superparamagnetic behavior of Fe magnetic moments in the studied samples. The relative intensity of the doubles depends on the kind of dopant. The most intensive doublet is observed for magnetite doped by Ca (more than 50%) and the smallest one in the case of Co dopant. These results show that the superparamagnetic blocking temperature for the Ca doping is below room temperature whereas for the other cases it is slightly above. Taking both features of the measured spectra into account, the mean values of the hyperfine field are equal to 43 T, 44 T, 39 T, 30 T and 20 T for pure magnetite and Co-, Ni-, Mn- and Ca-doped magnetite, respectively. This value strongly depends on the ferrite composition, particle size, surface modification and proximity to superparamagnetic blocking temperature.

### Attachment of Cu^2+^, Cd^2+^ and Pb^2+^ ions to unmodified nanoparticles

**Figure 7:**
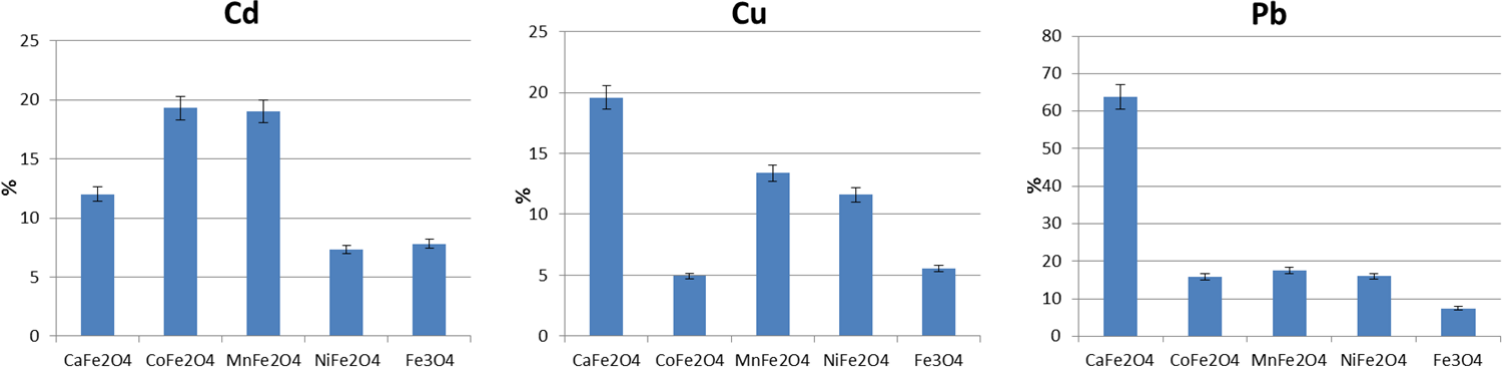
EDX measurements of different types of nanoparticles after exposure to Cd, Cu and Pb solutions.

In the first attempt, we have tested the effectiveness of attachment of metal ions to the unmodified surface of the nanoparticles. This test gives us a reference value and shows if inorganic core additional functionalization affects the interaction with ions. Therefore, ferrite nanoparticles were added to the aqueous heavy-metal solutions and stirred. The liquids were separated from the solid particles, which were dried at room temperature. Powdered samples after the adsorption experiment were examined by EDX and the results are given in Figure 7.

It can be seen that pure magnetite has the lowest capability of interaction with the tested ions. Mn_0.5_Fe_2.5_O_4_ and Co_0.5_Fe_2.5_O_4_ nanoparticles have a similar adsorption tendency of about 20% in case of Cd and Pb ions. Ni ferrite has an adsorption efficiency for Pb similar to that of the previous ferrites, but a very low adsorption efficiency for Cd^2+^. In case of Cu^2+^, the situation is different. Here, Mn_0.5_Fe_2.5_O_4_ and Ni_0.5_Fe_2.5_O_4_ show similar values of about 12%, while Co_0.5_Fe_2.5_O_4_ exhibits the worst performance. Interestingly, Ca ferrite adsorbs the most Pb^2+^ up to more than 60%. It seems that Mn_0.5_Fe_2.5_O_4_ has the most similar adsorption properties regarding all types of heavy metals. Hence, for further examination nanoparticles doped with Mn were used. Other ferrite particles will be a subject of further studies (not presented here).

### Adsorption of Cu, Cd, and Pb on nanoparticles with modified surfaces

Aqueous solutions of Cu, Cd, or Pb were prepared and added to the nanoparticles. The mixtures were vigorously mixed on a magnetic stirrer. Then, nanoparticles were removed and the concentrations of Cu, Cd, or Pb in the obtained solutions were analyzed using AAS. The data collected in Table 4 show the percentage values of adsorbed heavy-metal ions. It indicates that the best and most universal linker enhancing the adsorption of heavy-metal ions is SA. The PA linker is not selective for Cu ions, but rather good for Pb and Cd. We are aware that adsorption efficiency can be tuned by many other parameters, for example such as linker concentration, heavy ions concentration, pH value, or inorganic core composition, and such studies are in progress and will be a subject of subsequent papers.

**Table 4:** Percentage of ions adsorbed by Mn_0.5_Fe_2.5_O_4_ nanoparticles modified with different linkers measured by AAS. As a reference, unmodified nanoparticles were used.

	percentage of ions adsorbed		
	Pb	Cu	Cd

NPs	26.1 ± 0.2	22.9 ± 0.2	6.5 ± 0.2
NPs + PA	36.8 ± 0.2	3.2 ± 0.2	27.5 ± 0.2
NPs + SA	38.1 ± 0.2	23.8 ± 0.2	32.3 ± 0.2
NPs + AA	15.9 ± 0.2	14.4 ± 0.2	12.1 ± 0.2
NPs + 3-PPA	25.7 ± 0.2	12.3 ± 0.2	14.9 ± 0.2
NPs + 16-PHDA	1.9 ± 0.2	11.8 ± 0.2	7.6 ± 0.2

## Conclusion

Ferrite nanoparticles doped with calcium, cobalt, nickel, or manganese show differences in ion adsorption depending on the type of core. This indicates that the strength of the interparticle interaction can be one of the parameters governing the adsorption capability. It has been confirmed that calcium-modified ferrite is the most effective in Pb case, but only when the core is modified by PA. In any other case its role is average. IR measurements have proved a successful connection of the tested linkers to all nanoparticles, which entails that particles become a proper platform to further studies. AAS tests clearly confirmed the attachment of heavy metals to the selected nanoparticles (Mn_0.5_Fe_2.5_O_4_), with variable efficiency depending on the type of ions. SA appears to be the most effective and universal linker. The use of other types of compounds such as 3-PPA or 16-PHDA improved adsorption in selected cases, but they are not as versatile as succinic anhydride. Other anhydrides (PA and AA) also improve the adsorption of heavy metals in comparison to unmodified particles, but not as efficiently as SA. To conclude, succinic anhydride may be a good potential modifier of the Mn_0.5_Fe_2.5_O_4_ particles to enhance the selective adsorption of heavy metal ions from aqueous solutions. Therefore, in future, experiments with real food samples or a richer solution matrix will be performed. The adsorption efficiency strongly depends on core composition as well as surface modification, and only a complex analysis of the system allows one to draw adequate conclusions.
